# Effectiveness of Steroid Pulse Therapy for Systemic Side Effects after Bacillus Calmette-Guérin Intravesical Instillation Therapy: A Series of Five Cases

**DOI:** 10.1155/2021/5548054

**Published:** 2021-07-09

**Authors:** Tatsuya Umemoto, Jun Naruse, Yukio Usui, Hidenori Zakoji, Hideshi Miyakita, Akira Miyajima

**Affiliations:** ^1^Department of Urology, Tokai University Hachioji Hospital, 1838 Ishikawa-machi, Hachioji, Tokyo 192-0032, Japan; ^2^Department of Urology, Tokai University Oiso Hospital, 21-1, Gakkyo, Oiso-machi, Naka-gun, Kanagawa 259-0198, Japan; ^3^Department of Urology, Shizuoka City Shimizu Hospital, Miyakami 1231, Shimizu Ward Shizuoka City, Shizuoka 424-8636, Japan; ^4^Department of Urology, Tokai University School of Medicine, 143 Shimokasuya, Isehara, Kanagawa 259-1193, Japan

## Abstract

**Introduction:**

Bacillus Calmette-Guérin (BCG) instillation is an established therapy for the treatment of carcinoma in situ (CIS) of the bladder and prevention of recurrence after transurethral resection of bladder tumor noninvasive bladder cancer. However, serious systemic side effects may occur in less than 5% of patients with BCG intravesical instillation. Systemic side effects can sometimes be fatal and require early and accurate treatment. We describe five cases wherein steroid pulse therapy was effective for treating the systemic side effects after BCG intravesical instillation. *Case Presentations*. BCG intravesical instillation was used to prevent the recurrence of nonmuscle invasive bladder cancer and treat CIS of the bladder; the dose used was 40–80 mg each time, and the Tokyo strain was used. The patients developed fever, impaired consciousness, arthralgia, conjunctival hyperemia, and symptoms of cystitis. The median time from installation to side effect manifestation was 6 days (0–8). One to two courses of steroid pulse therapy were administered (1 course in 3 days), and the dose of methylprednisolone was 500–1000 mg/day. BCG sepsis was observed in one case; however, in the other four cases, one course of steroid pulse therapy showed a rapid improvement in symptoms. In the case of BCG sepsis, hemodialysis and mechanical ventilation were required because of septic shock and acute renal failure. Antituberculosis drugs (isoniazid, rifampicin, and ethambutol) were started promptly; however, no improvement was noticed. Two courses of steroid pulse therapy improved the patient's general condition, and hemodialysis and mechanical ventilation were no longer required. All patients survived without relapse of symptoms.

**Conclusion:**

Our cases suggest that early steroid pulse therapy may be effective for rapid symptom improvement of the systemic side effects of BCG instillation therapy.

## 1. Introduction

Bacillus Calmette-Guérin (BCG) instillation is an established therapy for the treatment of carcinoma in situ (CIS) of the bladder and prevention of recurrence after transurethral resection of bladder tumor (TURBT) for noninvasive bladder cancer [1–7]. BCG is an attenuated strain of *Mycobacterium bovis* that induces a cell-mediated immune response and induces attacks on malignant cells [8]. BCG infusion causes various side effects, such as symptoms of cystitis, nausea, fever, and hematuria; however, they are mostly mild. Additionally, it does have known severe side effects, including pneumonitis/hepatitis (0.7%), BCG sepsis (0.4%), and even death [9]. BCG infection is one of the side effects of BCG infusion therapy [10], which is classified into local infection, disseminated infection, and ectopic infection. The characteristics and treatment methods of each are shown in Table 1 [9]. Similarly, infection of aortic aneurysms has been reported as an ectopic infection [11]. In clinical practice, mild local side effects are most likely to improve rapidly with the discontinuation of BCG administration; however, systemic side effects are often difficult to treat.  Table 2 shows the management of side effects (local and systemic) after BCG perfusion therapy based on a partial excerpt from the European Association of Urology guidelines [11]. The guidelines show that systemic side effects include persistent high-grade fever (>38.5°C for >48 hours), arthralgia and/or arthritis, BCG sepsis, and allergic reactions. Nonsteroidal anti-inflammatory drugs (NSAIDs) are the first treatment choice for joint symptoms; however, if they are ineffective, the use of antituberculosis drugs and high-dose quinolones is recommended. Although antituberculosis drugs and quinolones are highly recommended for other systemic side effects, steroids are recommended for only BCG sepsis. We describe our experience of five cases wherein steroid pulse therapy was effective for the treatment of BCG instillation-induced systemic side effects.

## 2. Case Presentations

In all cases, BCG intravesical instillation therapy was started at six weeks or more after the operation.  Table 3 shows the characteristics of all patients. The median of the number of BCG therapy sessions performed before the manifestation of severe side effects was 4 (range, 3-8 sessions). According to Gonzales et al., stratification is proposed within three months from the first dose, and this likely applied to cases 1–4 due to the early presentation [10]. Case 5 was excluded because the patient received 14 courses of BCG maintenance therapy, and the side effect was caused by iatrogenic trauma.

### 2.1. Case 1

A 60-year-old woman underwent 80 mg BCG (Tokyo strain) intravesical instillation therapy for high-grade pTa bladder cancer. She presented with fever (>48 hours), knee joint pain, and conjunctival hyperemia after receiving four infusions. She was administered 10 mg/day of prednisolone (as per 0.2 mg/kg body weight), an NSAID (loxoprofen sodium hydrate, 180 mg/day), and antituberculosis drugs (isoniazid (INH), 300 mg/day; rifampicin (REP), 450 mg/day; and ethambutol (EB), 750 mg/day) because she was diagnosed with BCG-induced Reiter syndrome. She was administered steroid pulse therapy (methylprednisolone, 1000 mg/day) for three days because high white blood cell (WBC) and C-reactive protein (CRP) levels and fever persisted. After the third day of administration, fever, joint pain, and inflammation improved quickly. Subsequently, the dose of prednisolone was gradually reduced to a maintenance dose of 30 mg (as per 0.6 mg/kg body weight), and the symptoms did not recur.

### 2.2. Case 2

A 66-year-old woman underwent 80 mg BCG intravesical instillation therapy for bladder urothelial CIS. She presented with knee joint pain, conjunctival hyperemia, and urethritis after receiving eight infusions. She was administered prednisolone (30 mg/day as per 0.55 mg/kg body weight) and an NSAID (loxoprofen sodium hydrate, 180 mg/day) because she was diagnosed with BCG-induced Reiter syndrome. She was administered steroid pulse therapy (methylprednisolone, 1000 mg/day) for three days because high WBC and CRP levels persisted, and the symptoms were prolonged. After the third day of administration, the symptoms and inflammation improved quickly. Subsequently, the dose of prednisolone was gradually reduced to a maintenance dose of 30 mg (as per 0.55 mg/kg body weight), and the symptoms did not recur.

### 2.3. Case 3

A 76-year-old man underwent 80 mg BCG intravesical instillation therapy for high-grade pTa bladder cancer. He presented with fever (>48 hours), knee and elbow joint pain, and conjunctival hyperemia after receiving four infusions. He was administered an NSAID (loxoprofen sodium hydrate, 180 mg/day) and steroid pulse therapy (methylprednisolone, 500 mg/day) for three days because of high WBC and CRP levels and a diagnosis of BCG-induced Reiter syndrome. After the tenth day of administration, the symptoms and inflammation improved. Subsequently, the dose of prednisolone was gradually reduced to a maintenance dose of 30 mg (as per 0.5 mg/kg body weight), and the symptoms did not recur.

### 2.4. Case 4

A 77-year-old woman underwent 40 mg BCG intravesical instillation therapy for high-grade pT1 bladder cancer. She presented with knee joint pain and urethritis after receiving three infusions. She was administered an NSAID (celecoxib, 200 mg/day) for a few days. Subsequently, she was administered steroid pulse therapy (methylprednisolone, 1000 mg/day) for three days because she presented with fever (>48 hours), joint pain, and urethritis. After the second day of administration, her symptoms and inflammation quickly improved. After that, the dose of prednisolone was gradually reduced from 50 mg (1 mg/kg), and the symptoms did not recur.

### 2.5. Case 5

**Figure 1:**
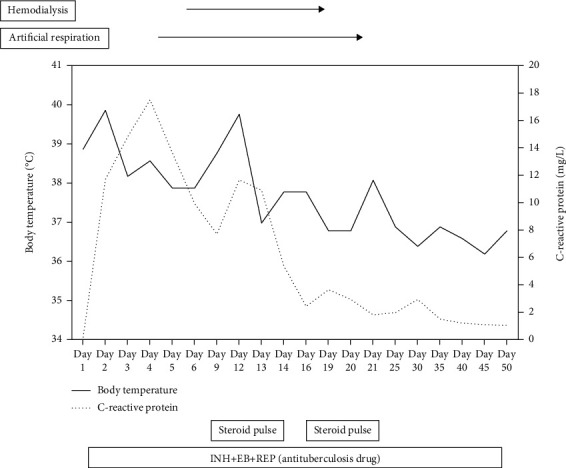
Treatment course of the patient with Bacillus Calmette-Guérin (case 5). EB = ethambutol; INH = isoniazid; REP = rifampicin.

A 73-year-old man underwent 80 mg BCG intravesical instillation therapy for high-grade pT1 bladder cancer. In addition, after receiving six infusions, he underwent BCG maintenance therapy every three months for three weeks, which was administered after using a urethral bougie to dilate his urethral stricture. On the same day, he was referred to our hospital because of fever and impaired consciousness. Upon examination, fever and decreased blood pressure were noted and the infected area was unclear; however, computed tomography (CT) revealed an increase in the perirenal fat concentration. BCG sepsis was diagnosed based on these findings and the patient's medical history of BCG administration after receiving the urethral bougie. The progress of treatment after hospitalization is shown in Figure 1. He was administered antituberculosis drugs (INH, 300 mg/day; RFP, 450 mg/day; and EB, 750 mg/day), an empirical nonspecific antibiotic, and an antidisseminated intravascular coagulation drug (thrombomodulin alfa) for BCG sepsis and disseminated intravascular coagulation after various tuberculosis tests were performed. Steroids were not administered because of the possibility of *M. bovis* infection. Ventilator management was performed for airway obstruction caused by oral bleeding, and dialysis management was required because of acute renal failure. Steroid pulse therapy was started because the fever persisted. CT on day 12 revealed findings suggestive of interstitial pneumonia. A diagnosis of interstitial pneumonia due to BCG was established based on the patient's presentations as follows: fever, decreased oxygenation, and no history of imaging findings of interstitial pneumonia. Steroid pulse therapy was administered (methylprednisolone, 1000 mg/day) for three days, and prednisolone was administered at a maintenance dose of 80 mg/day (as per 1 mg/kg body weight). The fever and inflammatory response reduced, and dialysis was no longer required. Additional steroid pulse therapy (methylprednisolone, 1000 mg/day for three days) was administered on day 19, and mechanical ventilation was discontinued on day 20. The dose of prednisolone was gradually reduced from 80 mg, and the symptoms did not recur. He was discharged on day 51. EB was stopped on day 64, and INH and REP were stopped on day 322; the symptoms have not recurred since then.

## 3. Discussion

In cases 1–4, fever (>48 hours) and arthralgia symptoms were commonly observed; however, fortunately, no severe side effects, such as BCG sepsis, were observed. Reiter syndrome was diagnosed in cases 1 to 4, and urethritis, conjunctivitis, and arthritis are reportedly known as the classic triad [12]. Incomplete Reiter's syndrome, which is not characterized by all three symptoms, as in case 1, has similarly been observed and is currently regarded as a subtype of reactive arthritis [13]. NSAIDs are primarily used for treatment; however, steroids are recommended in severe cases or when NSAIDs are inadequate [14].

Case 5 developed BCG sepsis and was the most severe case we experienced. The cause of these BCG-related complications remains controversial, and it is believed that the cause is a hypersensitivity inflammatory reaction or active infection due to *M. bovis* [15, 16]. It is difficult to prove the presence of *M. bovis* infection, and Perez-Jacoiste et al. reported that the microbiological-based diagnosis of *M. bovis* infection was positive in only 118 of 246 patients [10]. In case 5, the results of tests, including polymerase chain reaction, staining for acid-fast bacilli, and culture, for *M. bovis* infection were negative. In clinical practice, BCG sepsis can rapidly become severe, and often, treatment should be performed even in situations wherein test results are not available. Therefore, it is important to classify and memorize the information about therapeutic agents and start treatment as soon as possible if BCG sepsis is suspected.

Cystitis is the most common local side effect associated with BCG administration (80%) and is the most frequently cited reason for the postponement of BCG administration. Other local side effects include gross hematuria, atrophic bladder, and ureteral obstruction [17]. As shown in Table 1, local BCG infections include infections of the bladder, kidney, renal pelvis, ureter, prostate, and epididymis. First, it is recommended to postpone BCG administration, and if there is no improvement, surgical treatment and antituberculosis drugs should be considered. Pyrazinamide is not effective against *M. bovis*; hence, INH, RFP, EB, and streptomycin are used. Three-month administration of INH+RFP is recommended for local infections. Next, the management of BCG sepsis requires the cessation of BCG administration and examinations for *M. bovis* infection, including polymerase chain reaction, staining for acid-fast bacilli, and culture. A whole-body examination should be performed using imaging tests, such as CT, to evaluate the presence or absence of interstitial pneumonia and local *M. bovis* infection. Antituberculosis drugs (INH, RFP, and EB), a high-dose fluoroquinolone antibiotic, an empirical nonspecific antibiotic, and high-dose steroid therapy should be considered if there is no local infection. When interstitial pneumonia is observed, combination therapy with antituberculosis drugs and steroid therapy is often administered, and there are many reports of using pulse therapy as a steroid administration method [18]. The reason for the delay in the administration of steroid pulse therapy in case 5 was that there was a dissenting opinion that administration in a situation wherein the possibility of *M. bovis* infection could not be excluded was highly risky.

If steroid pulse therapy can be performed early, it is undeniable that it may have been relieved without becoming so severe. For urologists, steroid pulse therapy is not a routine treatment, and they may be hesitant to administer it owing to the associated side effects. According to a report on the side effects of steroid pulse therapy in other diseases, side effects (diabetes mellitus, moon face, hypertension, peptic ulcer, urinary tract infection, psychiatric symptoms, and susceptibility to infection, among others) are fewer with intravenous administration than with oral administration; however, severe liver damage may occur in 0.8% of the patients, and deaths were reported in 0.3%. The total recommended dose of methylprednisolone should be 8 g or less to reduce the risk of serious side effects [19–21]. Although high-dose steroids are described as one of the treatments for BCG sepsis, the dose and duration have not been clarified. Therefore, it seems that there are many cases wherein the effect is remarkable, as in our case; therefore, we think that early steroid pulse therapy for systemic side effects of BCG instillation is effective after monitoring for potential side effects.

BCG intravesical instillation therapy is one of the daily treatments for CIS of the bladder, and it is necessary to understand the associated side effects and be familiar with their management. To prevent side effects, the administration should be performed at least two weeks after TURBT and postponed for patients with gross hematuria, trauma due to catheter insertion, or urinary tract infection [22]. In case 5, the patient was administered BCG therapy after using a urethral bougie to dilate the urethral stricture. Since the patient developed an iatrogenic side effect caused by injecting BCG on the same day, we think that sufficient caution and consideration were required. BCG is thought to elicit a cell-mediated immune response and induce an attack on malignant cells [8]. The cause of BCG-related systemic side effects is supposedly a hypersensitivity inflammatory reaction or active infection due to *M. bovis* [16, 17]. One of the causes of Reiter syndrome after BCG administration is considered to be mediated by an immune response, including a lymphocyte T cell response [23]. Therefore, steroid administration, especially pulse therapy, is considered effective to strongly suppress the immune response. Steroid pulse therapy should be performed for Reiter syndrome or Reiter syndrome with fever when there are no locally infected lesions and in cases without sepsis or organ damage. Therefore, high-dose fluoroquinolones, antituberculosis drugs, an empirical nonspecific antibiotic, and early steroid pulse therapy should be considered for BCG sepsis.

## 4. Conclusions

We described five cases wherein steroid pulse therapy was used to treat systemic side effects after BCG intravesical instillation therapy. Our cases suggest that steroid pulse therapy may be important for early recovery from the systemic side effects after BCG intravesical instillation therapy in the absence of local infection. Additionally, in BCG sepsis, it is important to always consider not only antituberculosis drugs but also steroid pulse therapy as a treatment option.

## Figures and Tables

**Table 1:** Types of Bacillus Calmette-Guérin infections and treatments.

	Type of BCG infection	Treatment
Local	Infection of the bladder, kidney, renal pelvis, ureter, prostate, and epididymis.	Discontinue BCG. Administer isoniazid, rifampicin, and ethambutol for 3–6 months.
Systemic	Hematogenous dissemination of BCG to the systemic organs (BCG sepsis, hepatitis, and cerebromeningitis, among others).	Discontinue BCG. Administer isoniazid, rifampicin, and ethambutol for six months.
Ectopic	Ectopic infection of aneurysms and artificial organs (artificial joints, etc.), among others.	Administer isoniazid and rifampicin for one year. Consider excision of the infected area.

BCG = Bacillus Calmette-Guérin.

**Table 2:** Management options for treating the side effects of Bacillus Calmette-Guérin intravesical instillation therapy.

Management options for local side effects	
Symptomatic granulomatous prostatitis	Symptoms rarely present: perform urine culture.
	Administer quinolones.
	If quinolones are not effective, administer isoniazid (300 mg/day) and rifampicin (600 mg/day) for three months.
	Stop intravesical instillation therapy.

Epididymo-orchitis	Perform urine culture and administer quinolones.
	Stop intravesical instillation therapy.
	Perform orchidectomy if an abscess is present or if there is no response to treatment.

Management options for systemic side effects	
General malaise, fever	These generally resolve within 48 hours, with or without antipyretics.

Arthralgia and/or arthritis	This is a rare complication and is considered an autoimmune reaction.
	Arthralgia: treat with NSAIDs.
	Arthritis: treat with NSAIDs.
	If no/partial response, proceed to treatment with corticosteroids, high-dose quinolones, or antituberculosis drugs.

Persistent high-grade fever (>38.5°C for >48 h)	Permanently stop BCG instillation.
	Immediate evaluation: urine culture, blood tests, chest X-ray.
	Administer prompt treatment with more than two antimicrobial agents while a diagnostic evaluation is conducted.
	Consult with an infectious disease specialist.

BCG sepsis	Prevention: initiate BCG at least two weeks after transurethral resection of the bladder (if there are no signs and symptoms of hematuria).
	Stop BCG instillation.
	For severe infection: Administer high-dose quinolones or isoniazid, rifampicin, and ethambutol (1.2 g daily) for six months. Administer early, high-dose corticosteroids as long as symptoms persist. Consider administering an empirical nonspecific antibiotic to cover Gram-negative bacteria and/or *Enterococcus.*

BCG = Bacillus Calmette-Guérin; NSAIDs = nonsteroidal anti-inflammatory drugs.

**Table 3:** Patient characteristics.

	Age (y)	Sex	Bladder cancer stage/type	Number of BCG therapy sessions	Side effect	Antituberculosis drug	Steroid pulse therapy (type, dose; duration)	Outcome
Case 1	60	Female	pTa high-grade	4	Fever, knee joint pain, conjunctival hyperemia	INH, 300 mg/day; REP, 450 mg/day; EB, 750 mg/day	Methylprednisolone, 1000 mg/day; 3 days (1 course)	Alive
Case 2	66	Female	CIS	8	Knee joint pain, conjunctival hyperemia, urethritis	None	Methylprednisolone, 1000 mg/day; 3 days (1 course)	Alive
Case 3	76	Male	pTa high-grade	4	Fever, knee and elbow joint pain, conjunctival hyperemia	None	Methylprednisolone, 500 mg/day; 3 days (1 course)	Alive
Case 4	77	Female	pT1 high-grade	3	Fever, knee joint pain, urethritis	None	Methylprednisolone, 1000 mg/day; 3 days (1 course)	Alive
Case 5	73	Male	pT1 high-grade	14	Fever, impaired consciousness, BCG sepsis	INH, 300 mg/day; REP, 450 mg/day; EB, 750 mg/day	Methylprednisolone, 1000 mg/day; 6 days (2 courses)	Alive

BCG = Bacillus Calmette-Guérin; INH = isoniazid; REP = rifampicin; EB = ethambutol; CIS = carcinoma in situ.
